# *Lactobacillus mucosae* DPC 6426 as a bile-modifying and immunomodulatory microbe

**DOI:** 10.1186/s12866-019-1403-0

**Published:** 2019-02-08

**Authors:** Paul M. Ryan, Ellen H. Stolte, Lis E. E. London, Jerry M. Wells, Sarah L. Long, Susan A. Joyce, Cormac G. M. Gahan, Gerald F. Fitzgerald, R. Paul Ross, Noel M. Caplice, Catherine Stanton

**Affiliations:** 1 Teagasc Food Research Centre, Food Biosciences Department, Moorepark, Fermoy, Co, Cork, Ireland; 2 School of Microbiology, University College Cork, Cork, Ireland; 3 Host–Microbe Interactomics, University of Wageningen, Animal Sciences Department, Wageningen, The Netherlands; 4 APC Microbiome Ireland, University College Cork, Cork, Ireland; 5 School of Biochemistry and Cell Biology, University College Cork, Cork, Ireland; 6 School of Pharmacy, University College Cork, Cork, Ireland; 7 Centre for Research in Vascular Biology, University College Cork, Cork, Ireland

**Keywords:** Exopolysaccharide, Bile acid, Bile salt hydrolase (BSH), Hypercholesterolaemia, CVD

## Abstract

**Background:**

*Lactobacillus mucosae* DPC 6426 has previously demonstrated potentially cardio-protective properties, in the form of dyslipidaemia and hypercholesterolemia correction in an apolipoprotein-E deficient mouse model. This study aims to characterise the manner in which this microbe may modulate host bile pool composition and immune response, in the context of cardiovascular disease. *Lactobacillus mucosae* DPC 6426 was assessed for bile salt hydrolase activity and specificity. The microbe was compared against several other enteric strains of the same species, as well as a confirmed bile salt hydrolase-active strain, *Lactobacillus reuteri* APC 2587.

**Results:**

Quantitative bile salt hydrolase assays revealed that enzymatic extracts from *Lactobacillus reuteri* APC 2587 and *Lactobacillus mucosae* DPC 6426 demonstrate the greatest activity in vitro. Bile acid profiling of porcine and murine bile following incubation with *Lactobacillus mucosae* DPC 6426 confirmed a preference for hydrolysis of glyco-conjugated bile acids. In addition, the purified exopolysaccharide and secretome of *Lactobacillus mucosae* DPC 6426 were investigated for immunomodulatory capabilities using RAW264.7 macrophages. Gene expression data revealed that both fractions stimulated increases in interleukin-6 and interleukin-10 gene transcription in the murine macrophages, while the entire secretome was necessary to increase CD206 transcription. Moreover, the exopolysaccharide elicited a dose-dependent increase in nitric oxide and interleukin-10 production from RAW264.7 macrophages, concurrent with increased tumour necrosis factor-α secretion at all doses.

**Conclusions:**

This study indicates that *Lactobacillus mucosae* DPC 6426 modulates both bile pool composition and immune system tone in a manner which may contribute significantly to the previously identified cardio-protective phenotype.

**Electronic supplementary material:**

The online version of this article (10.1186/s12866-019-1403-0) contains supplementary material, which is available to authorized users.

## Background

The gut microbiome is a complex ecosystem of diverse metabolic pathways which is central to the progression and prevention of host cardiovascular disease (**CVD**) and other closely related metabolic dysfunctions such as obesity and type-2 diabetes [1]. These microorganisms are capable of contributing specifically to atherogenesis through a number of pathways, with the potential to alter gut hormone signalling, bile and lipid metabolism and inflammatory status [2]. Conversely, under the correct environmental conditions such as favourable nutrition, certain members of the gut microbiome can actively counteract the factors which contribute to atherogenesis [3].

In clinical terms, plasma lipid profiles are among the most important predictors for cardiovascular dysfunction. The sizeable human population which are classified as dyslipidaemic rely heavily on statins for disease prevention and management. However, recent insight has demonstrated that certain members of the enteric microbiota can also have beneficial effects on host lipid metabolism, with significant knock-on implications for cardiovascular function [4]. *Lactobacillus mucosae* DPC 6426 has been shown to attenuate the dyslipidaemia and hypercholesterolemia observed in apolipoprotein-E deficient mice maintained on a high-fat/cholesterol diet [5], and may have application as a potential therapy or adjunct to pharmaceutical CVD intervention. One mechanism through which gut microbes are known to impact on host lipid profile is through a set of enzymes, termed bile salt hydrolases (**BSH**), which function in deconjugating bile salt to bile acid (**BA**) [6]. These enzymes are produced primarily as a defence against the harsh enteric environment and act in cleaving the amino group off a BA, rendering the molecule amenable to further degradation by other bacterial enzymes, such as 7-α-dehydroxylases [7–9]. This process reduces BA reabsorption in the ileum and in turn can up-regulate de novo synthesis of BA, of which cholesterol is a major component. This effect is the result of the suppression of the ileal farnesoid X receptor-fibroblast growth factor (**FXR-FGF)**15/19 axis, which impacts through the hepatocyte membrane FGFR4/ß-klotho complex primarily on CYP7A1-expression downstream in the cascade; ultimately, improving the host lipid profile and metabolic health [10, 11], potentially leading to a reduced risk of CVD.

A second means through which gut bacteria may alter host lipid metabolism is through expression of complex polysaccharides, termed exopolysaccharides (**EPS**). A previous study from our group has demonstrated the ability of *Lactobacillus mucosae* DPC 6426 – a natural EPS^+^ strain – and a recombinant EPS^+^*Lactobacillus* spp. to alter lipid metabolism in an apolipoprotein-E deficient mouse model [5]. In this study, *Lactobacillus paracasei* NFBC 338 was transformed to express the glycosyltransferase gene of *Pediococcus parvulus* 2.6 in the pNZ44 plasmid. This strain was compared against its EPS^−^ isogenic control (solely expressing the empty plasmid vector) for their effects on host lipid profile. The EPS^+^ strains demonstrated the ability to significantly reduce host serum cholesterol and triglyceride levels by up to 50 and 25%, respectively, while also modulating the effects of several proinflammatory and proatherogenic factors - however the mechanisms underlying these improvements have not been entirely determined.

Inflammation plays a central role in the development and progression of several metabolic disease states [12], including those which lead to atherosclerosis [13]. One major contributor to this process is inflammatory lipopolysaccharide (**LPS**)-mediated Toll-like receptor (**TLR**)4 signalling, a process which has a substantial negative impact on atheroprotective reverse cholesterol transport. TLR4-activation suppresses liver X receptor expression [14, 15], which in turn results in increased low-density lipoprotein receptor, very low-density lipoprotein receptor and adiponectin receptor-2 transcription [16] – ultimately promoting lipid accumulation in macrophages, which results in foam cell formation and atherogenesis. Conversely, immunological pathways exist through which the gut microbiome may inhibit inflammation and other immunological pathologies associated with CVD and metabolic dysfunction. Interleukin (**IL**)-10, a potent anti-inflammatory molecule which can be stimulated by commensal and probiotic metabolites such as EPS [17], has displayed a protective role in the development of atherosclerosis. In addition, several prebiotics [18] and probiotics [19] have previously displayed considerable potential to attenuate host inflammation in a useful manner and it is likely that such an effect will have implications for metabolic health and, in turn, CVD risk. Indeed, co-culture of human macrophages with the koumiss-derived *Lactobacillus helveticus* NS8 has been demonstrated to induce IL-10 secretion and attenuate the synthetic inflammation created in a murine model of colitis [20]. Moreover, a kimchi-isolated strain of *Lactobacillus brevis* has also been found to impact beneficially upon the development of experimental colitis in mice through increased polarization of alternatively activated macrophage and inhibition of the nuclear factor-κB (**NF-κB**) pathway [21]. Interestingly, the application of a heat-killed *Lactobacillus plantarum* to an animal model of obesity and insulin resistance revealed an immunomodulatory and lipid metabolism altering effect of the strain [22]. This suggests that such attributes may be mediated by a structural molecule, such as EPS. This study aims to assess various properties of *Lactobacillus mucosae* DPC 6426 which might mechanistically address the ability of the strain to exhibit cardio-protective effects as observed in mice studies.

## Results

### *Lactobacillus mucosae* DPC 6426 secretome and EPS induce macrophage phenotypes that share features of both classically and alternatively activated macrophages

**Fig. 1:**
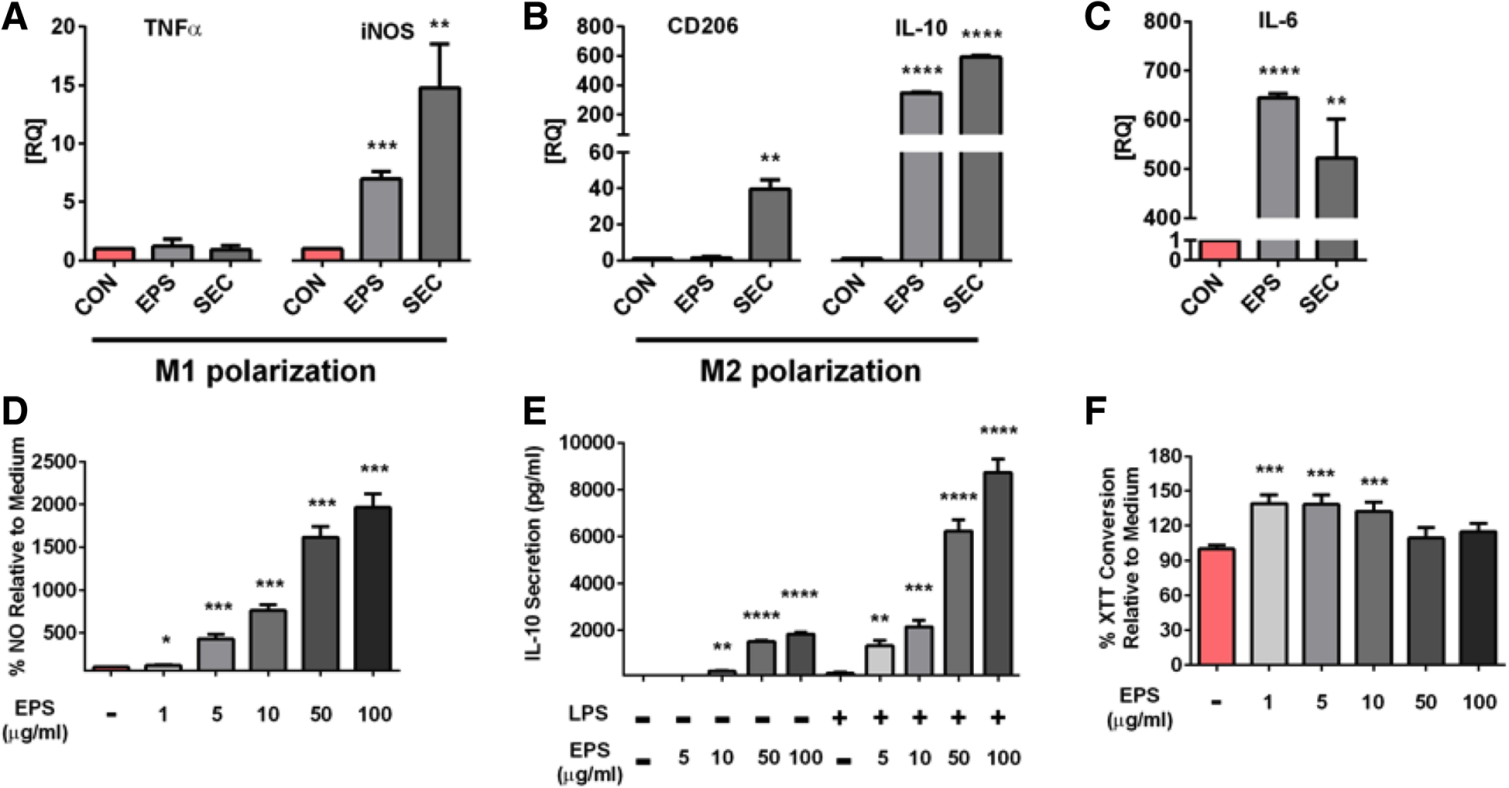
*Lactobacillus mucosae* DPC 6426 Macrophage Polarization. Transcription of M1-related markers (**a**) TNF-α and iNOS, M2-related markers (**b**) CD206 and IL-10, as well as (**c**) IL-6 following 24 h exposure to either the secretome of *Lactobacillus mucosae* DPC 6426 (**SEC**) or 10 mg/mL of isolated EPS. Results for the same markers are also displayed for polarised macrophages. In a separate series of experiments, macrophages were stimulated with EPS for 24 h with or without additional LPS stimulation after which NO (**d**) and IL-10 (**e**) production was measured. In the same cells, viability was measured using an XTT assay (f). All experiments were conducted three times with duplicate samples. Data are shown as average ± SD, and were analysed by one-way ANOVA. Differences were considered statistically significant when *p* < 0.05 (*), *p* < 0.01 (**) or *p* < 0.001 (***)

Recently, it was posited that ingestion of *Lb. mucosae* DPC 6426 alters lipid metabolism due to the production of a complex polysaccharide [5]. To investigate the ability of EPS isolated from *Lb. mucosae* DPC 6426 and the secretome of *Lb. mucosae* DPC 6426 to induce macrophage polarization – a key factor in the inflammation-mediated development of atherosclerosis – alterations in tumor necrosis factor (**TNF**)-α, inducible nitric oxide synthase (**iNOS**), CD206, IL-6 and IL-10 gene expression were analysed in a murine macrophage cell line. Stimulation with LPS/interferon-gamma (**IFN-γ**) or IL-4 was included in each experiment to ensure functional differentiation into M1 or M2 subtypes, respectively. The average relative increase in TNF-α and iNOS transcription after LPS/IFN-γ treatment was 5-fold and 2.5-fold, respectively, while the average increase in CD206 and *Il-10* gene expression after IL-4 treatment was 40-fold and 300-fold, respectively – indicating successful polarization (data not shown). Neither the secretome nor isolated EPS from *Lb. mucosae* DPC 6426 significantly increased gene expression of the inflammatory cytokine TNF-α (Fig. 1A). In contrast, relative expression of the iNOS gene, which is expressed in classically activated macrophages, was increased by both the *Lb. mucosae* EPS and secretome fractions compared to unstimulated cells (*p* < 0.01; Fig. 1A). Interestingly, gene expression for CD206 following stimulation with the secretome of *Lb. mucosae* DPC 6426 was significantly increased (*p* < 0.01), while the isolated EPS stimulation had no such effect (Fig. 1B). IL-10 gene expression, a marker of alternatively activated macrophages was significantly increased following stimulation with both the secretome and isolated EPS of *Lb. mucosae* DPC 6426 (*p* < 0.0001; Fig. 1B). The gene expression for IL-6 was significantly increased following stimulation with the secretome (*p* < 0.01) and EPS (*p* < 0.0001), compared to unstimulated macrophages (Fig. 1C). Thus, the *Lb. mucosae* secretome and EPS induce macrophage phenotypes that share features of both classically and alternatively activated macrophages and in particular the anti-inflammatory cytokine IL-10.

### Macrophage stimulation with EPS induces relatively low amounts of NO compared to LPS

Stimulation of RAW 264.7 macrophages with 1 μg/ml EPS did not increase NO production above basal levels in unstimulated cells (1–2 uM) and 5 μg/ml EPS increased NO production to approximately 5 uM. In contrast, stimulation of macrophages with 1 μg/ml LPS increased NO production to 25–50 μM, which was only achieved by stimulation with large quantities (100 μg/ml) of EPS (Fig. 1D). LPS induced NO production was moderately increased by addition of 10 μg/ml or more EPS in a dose-dependent fashion. Stimulation of macrophages with 1μg/ml LPS or low amounts of EPS (1–10 μg/ml) increased the cellular metabolic activity whereas the combination of 1μg/ml LPS with 10 to 100 μg/ml EPS decreased metabolic activity despite the greater amount of induced NO production. These results indicate that relatively large amounts of EPS are required to induce more than 5 uM NO production in unstimulated macrophages.

### *Lactobacillus mucosae* DPC 6426 EPS does not affect oxidative burst activity

Isolated EPS did not affect the oxidative burst activity in neutrophils (Additional file 1: Figure S1), and this was demonstrated with neutrophils differentiated from a human cell line, as well as with freshly isolated neutrophils obtained from healthy volunteers. Moreover, even in neutrophils activated by PMA, addition of isolated EPS did not increase the production of reactive oxygen species (**ROS**). Again, this result was found for neutrophils that were derived from a cell line and freshly isolated neutrophils alike.

### *Lactobacillus mucosae* DPC 6426 is highly BSH-active

**Fig. 2:**
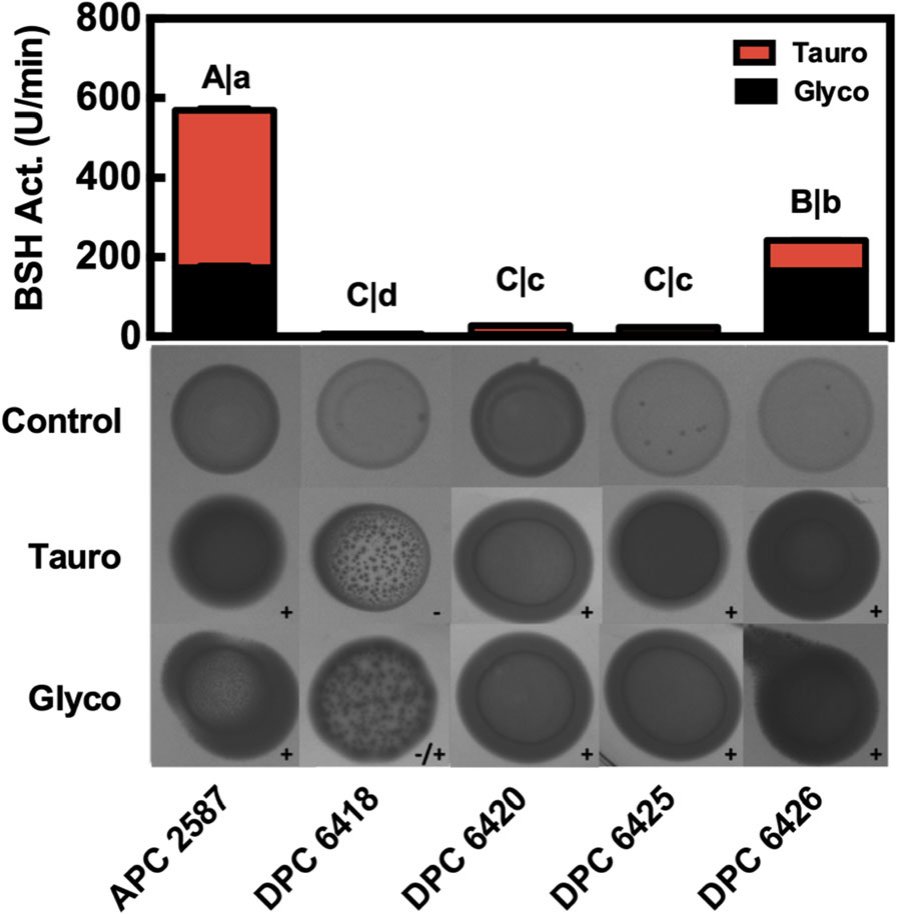
Bile Salt Hydrolase Activity of *Lactobacillus mucosae* Strains Compared to a Bile Deconjugating *Lactobacillus reuteri*. One unit of bile salt hydrolase activity is defined here as nmol of amino acid (taurine or glycine) cleaved from the relevant bile acid per mg of protein per minute. Taurine and glycine bile salt hydrolase activity are portrayed with red and black bars, respectively. Bars represent the means of triplicate experiments and error bars represent standard deviations. Images depict cell masses of each strain grown on MRS containing no bile acids (Control), MRS supplemented with taurodeoxycholic acids (Tauro) and glycodeoxycholic acid (Glyco). Strains which demonstrate bile salt hydrolase activity towards a particular bile acid are shown with a positive sign (+), while lack of activity is shown with a negative sign (−). Weak activity is indicated by +/−

Plate assays (Fig. 2) revealed that all strains introduced previously had some level of BSH activity for glycodeoxycholic acid (**GDCA**), while only *Lb. mucosae* DPC 6418 showed no apparent deconjugation activity for taurodeoxycholic acid (**TDCA**). The largest halo was recorded for *Lb. mucosae* DPC 6426 grown on TDCA-supplemented MRS; while the weakest halo was seen in *Lb. mucosae* DPC 6418 grown on GDCA-supplemented MRS. BSH activity assays performed with standardised protein extracts demonstrated that all strains bar *Lb. mucosae* DPC 6418 showed greater affinity to deconjugate GDCA than TDCA (*p* < 0.001). *Lb. reuteri* APC 2587 showed the greatest BSH activity for both TDCA (*p* < 0.0001) and GDCA (*p* < 0.0001) at 174 and 395 U min^− 1^, respectively. *Lb. mucosae* DPC 6426 followed closely with 75 and 165 U min^− 1^ for TDCA and GDCA, respectively. Both *Lb. mucosae* DPC 6420 and 6425 had similar, but greatly reduced GDCA BSH activity of ~ 20 U min^− 1^, which was still significantly higher than that of *Lb. mucosae* DPC 6418 (~ 5 U min^− 1^; *p* < 0.001). Finally, *Lb. mucosae* DPC 6420, 6425 and 6418 all demonstrated < 6 U min^− 1^ of TDCA BSH activity, which was significantly lower than that of both *Lb. reuteri* APC 2587 and *Lb. mucosae* DPC 6426 (*p* < 0.0001).

### *Lactobacillus mucosae* DPC 6426 displays preference for glyco-conjugated bile acids

**Fig. 3:**
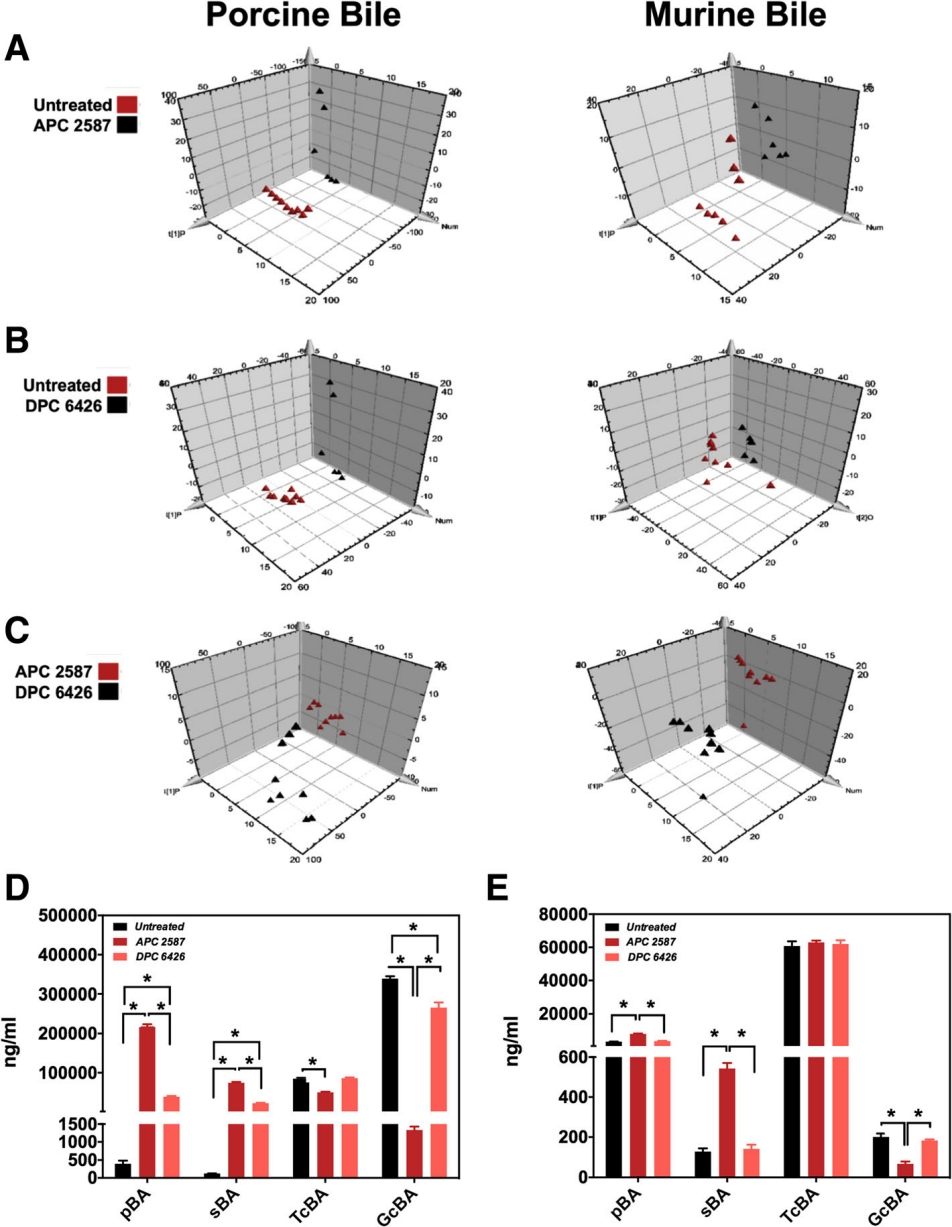
Deconjugation Profile of Porcine and Murine Bile Supplemented Culture Media Incubated with *Lactobacillus mucosae* DPC 6426 or a Bile Salt Hydrolase Active *Lactobacillus reuteri*. Principal component analysis (**PCA**) plots of bile deconjugation profiles of the two most active bile salt hydrolase strains in this study, *Lb. mucosae* DPC 6426 and *Lb. reuteri* APC 2587, are compared against untreated bile (**a** and **b,** respectively) and each other (**C**). Porcine (**d**) and murine (**e**) bile supplemented MRS bile acid profiles untreated (black), following overnight incubation with *Lb. reuteri* APC 2587 (dark red) or *Lb. mucosae* DPC 6426 (light red). Data are divided into primary bile acids (***pBA***), secondary bile acids (***sBA***), tauro-conjugated bile acids (***TcBA***) and glyco-conjugated bile acids (***GcBA***). Data were considered statistically significant when *p* < 0.05 (*) and plots depict replicates with mean and SEM

Of the 25 BAs quantified in this assay, 21 were found to be significantly altered in porcine bile following incubation with *Lb. reuteri* APC 2587 (Additional file 1: Table S2; *p* < 0.05). Of the remaining four unaltered BAs, three were not detected at any level in the porcine bile. Co-incubation of *Lb. reuteri* APC 2587 with murine bile resulted in alterations of 13 BAs (*p* < 0.05), principally primary BAs (***pBA***) and tauro-conjugated BAs (***TcBA***; Fig. 3E). Conversely, incubation with *Lb. mucosae* DPC 6426 significantly altered 13 BAs in porcine bile and just three in murine bile (*p* < 0.05) – taurolithocholic acid (**TLCA**), deoxycholic acid (**DCA)** and glycohyodeoxycholic acid (**GHDCA)**. When the bile profiles were grouped into their subcategories (Fig. 3D/E), it became clear that *Lb. mucosae* DPC 6426 favoured glyco-conjugated BA (***GcBA***) hydrolysis and, in turn, significantly increased both *pBA* and secondary BA (***sBA***) levels (*p* < 0.05). *Lb. reuteri* APC 2587 also displayed a greater affinity for *GcBA*-hydrolysis, however, the strain also proved capable of depleting murine bile *TcBA* (*p* < 0.05). Principal component analysis (**PCA**) plots of the data demonstrate that both strains altered porcine and murine bile to such a degree that the data points cluster distinctly from the untreated bile (Fig. 3A/B). However, it is apparent that the impact of *Lb. mucosae* DPC 6426 on the murine bile composition was modest. When both strains are plotted together, we observe distinct clustering of samples by treatment, giving further indication that the two *Lactobacillus* spp. demonstrate specificity in their BA hydrolysis activity and distinct substrate preferences.

## Discussion

CVD, and the debilitating metabolic dysfunctions which so often accompany it, are a global problem with few effective, non-invasive therapies free of adverse side-effects. Moreover, a significant portion of the at-risk population can be classed as mildly hypercholesterolemic. This cohort has been proven to benefit significantly from cholesterol-reducing bacterial therapies, without significant adverse events [23, 24]. *Lb. mucosae* DPC 6426 has demonstrated the ability to attenuate circulating cholesterol accumulation in a murine model of high-fat diet-aggravated CVD [5]. However, the mechanism through which this microorganism exerts its cardio-protective effects has remained unclear to date. In this study, we aimed to explore two notable metabolic features of the strain – the EPS and bile-deconjugating enzymes which it produces – and, ultimately, to determine the potential implications for host cardiometabolic health.

Classically activated macrophages, defined as the M1 subtype, are characterized by the expression of high levels of TNF-α*,* IL-6 and iNOS, and therefore amplify inflammation. Alternatively activated macrophages, known as the M2 subtype, promote tissue homeostasis by releasing IL-10 and transforming growth factor-β and rely heavily on β-oxidation [25]. The two subsets are present in human atherosclerotic plaque development [26, 27], with atherosclerotic lesions being characterized by macrophages with the M1 phenotype. Both the *Lb. mucosae* secretome and EPS induced macrophage phenotypes share features of both classically and alternatively activated macrophages. A notable feature of EPS and secretome stimulation of macrophages was the transcription of anti-inflammatory cytokine IL-10 and the scavenger receptor CD206, which are both characteristic of anti-inflammatory macrophages. Additionally, we showed that EPS could increase IL-10 production in LPS activated macrophages in a dose dependent fashion. Interestingly, both fractions of *Lb mucosae* increased IL-6 expression in macrophages. IL-6 is the most abundant cytokine and has frequently been uncovered in the context of obesity-associated metabolic disorders [28, 29]. However, it was recently demonstrated that IL-6 may control the maintenance of M2 macrophages [30]. IL-6, similar to IL-10 is a potent inducer of the IL-4/STAT6 axis which is essential for M2 macrophage polarization. Thus, IL-6 may exert its effect as a priming factor for anti-inflammatory IL-4 action in macrophages. Although iNOS transcription (a marker of M1 phenotype) was increased in EPS and secretome stimulated macrophages, EPS was not a strong inducer of NO at low concentrations. Furthermore, the EPS of *Lb. mucosae* DPC 6426 did not induce ROS production in HL60 cells differentiated into neutrophil-like cells or freshly isolated neutrophils (Supplementary Fig. 1).

Taken together, these results suggest that *Lb. mucosae* DPC 6426 in combination with its secreted metabolites including the EPS could negatively impact on atherogenesis via the effect of its anti-inflammatory activities in the intestinal lamina propria and mucosal lymphoid tissues. Intestinal immunity has attracted much attention as a therapeutic target for atherosclerosis and other inflammatory diseases [31]. Heightened inflammation in the intestine is linked to increased epithelial permeability and increased levels of LPS in the tissues and blood. Translocation of small numbers of bacteria or bacterial products such as LPS is now regarded as one important mechanism causing the low-grade inflammation in the liver and other tissues [12, 32]. In the lymphoid tissues, the anti-inflammatory properties of *Lb. mucosae* DPC 6426 could support the induction of tolerogenic responses in gut macrophages and dendritic cells supporting the integrity of the gut barrier and promoting induction of regulatory T cells, which have been proposed to be atheroprotective [33].

It is relevant at this point to note that the EPS produced by *Lb. mucosae* DPC 6426 is unusual for that of its genus. In fact, the heteropolysaccharide is reported to contain a high proportion of mannose residues in addition to the more common glucose and galactose. Importantly, mannose residues are more commonly associated with pathogenic or opportunistic organisms and, as a result, there are a number of human carbohydrate receptors which may initiate an immune response when faced with such a ligand. Such receptors include, but are not limited to, DC-SIGN, Dectin-2, MBL-1, MBL-2, CD206, CD280 and Mincle. For instance, the cell membranes of yeasts are coated densely with a mannoprotein-polysaccharide complex which could potentially act on the same receptors as the EPS described in this study. In the case of yeast, the polysaccharide zymosan is phagocytised following activation of macrophage CD206 (mannose receptor), after which internal TLR2 activation results in increased TNF-α secretion [34]. It is indeed likely that the unusual structure of the EPS plays a role in the expression of IL-10 in macrophages.

BSH activity assays revealed that both *Lb. reuteri* APC 2587 and *Lb. mucosae* DPC 6426 protein extracts contained one or more BSH homologues at impactful levels, while the other three *Lb. mucosae* displayed only modest or negligible BSH activity. *Lb. mucosae* DPC 6418, the strain which demonstrated the least BSH activity, also failed to form a full cell mass on plate assays (Fig. 2). This suggests that it may not actually be adapted to the bile-rich enteric environment, despite its reported enteric origin (Additional file 1: Table S1). The BSH activity results in fact mirror those of the bile profiling, in that *Lb. reuteri* APC 2587 had affinity for both *GcBA* and *TcBA* substrates, while *Lb. mucosae* DPC 6426 displayed similar *GcBA*-hydrolysis, but relatively reduced *TcBA*-hydrolysis. Several different *Lactobacillus* spp. have previously been reported to show a substrate preference towards *GcBA* over *TcBA*-hydrolysis [35, 36].

BAs have emerged as signalling molecules that interact with specific cellular receptors to regulate host metabolism [2, 37, 38]. Due to the central role of the gut microbiome in profoundly shaping the diversity of the systemic bile pool, BA offer an intriguing and convincing pathway for crosstalk between gut microbes and their host. Interestingly, a study from the Bäckhed group elegantly revealed that the altered bile profile observed in germ-free mice may contribute to their altered metabolism by affecting the gut microbiome [39]. This form of two-way communication is relatively rare in microbe-host dialogue. Sayin et al. [37] demonstrated the importance of tauro-conjugated muricholic acids (i.e. tauro-β-muricholic acid) as FXR antagonists in the murine system, while chenodeoxycholic acid (**CDCA**) demonstrates the greatest agonistic efficacy [40, 41]. Activation of FXR receptors in turn regulates FGF-15/19 and CYP7A1 expression, with implications for several metabolic pathways in the host hepatocytes including those involved in cholesterol metabolism [42].

At a simplistic level, microbial-mediated BA modification increases their hydrophobicity, thereby leading to decreased reabsorption and increased excretion of cholesterol-rich BAs in the faeces [43]. Indeed, intervention with *Lactobacillus* spp. in metabolically dysfunctional rodents has previously been shown to increase faecal BA excretion and ameliorate the lipid profile [44]. By reducing the reabsorption of BA and creating a low transhepatic flux, there is an increase in CYP7A1 transcription that leads to a reduction in hepatic LDL-C levels as a result of their use in BA synthesis [45]. Both *Lb. reuteri* APC 2587 and *Lb. mucosae* DPC 6426 exhibited considerable effects on CDCA in porcine bile, increasing the BA > 400-fold and > 350-fold, respectively. However, in vivo these newly unconjugated BA become open to further catabolism by strains expressing alternate enzymes [46]. This suggests that both strains could alter host bile in humans in a manner that would promote BA synthesis and cholesterol excretion. Interestingly, although *Lb. reuteri* APC 2587 and *Lb. mucosae* DPC 6426 both demonstrated significant activity against both porcine and murine bile, neither proved capable of reducing TcMCA levels. This could be in part explained due to their respective porcine and bovine origins (Additional file 1: Table S1), in which they would not be exposed to murine BA.

Although the FXR-FGF15/19 axis is of central importance in the effects of BAs on host lipid metabolism, it should be noted that BA receptors have also been shown to trigger cascades which directly impact on cardiovascular function without necessarily regulating lipid metabolism. There are several G-protein coupled receptors involved in the effects of BAs on host metabolism, such as TGR5 and muscarinic receptors. TGR5 is activated by lithocholic acid, TLCA, cholic acid (**CA**), deoxycholic acid (**DCA**) and CDCA, all of which were found to be significantly increased in porcine bile following incubation with either *Lb. mucosae* DPC 6426 or *Lb. reuteri* APC 2587, bar TLCA which was decreased. While TGR5 has been shown to impact directly upon atherogenic inflammation though cAMP-mediated secretion of NO [47] and inhibition of macrophage NF-κB signalling [48], it has also been shown to modulate glucagon-like peptide (**GLP**)-1 secretion [49]. Crucially, recent evidence indicates that increasing GLP-1 levels can have significant indirect knock-on effects for enterocyte formation of triglyceride-rich chylomicrons [50, 51], while also directly eliciting hypotensive atrial natriuretic peptide release from cardiomyocytes [52].

**Fig. 4:**
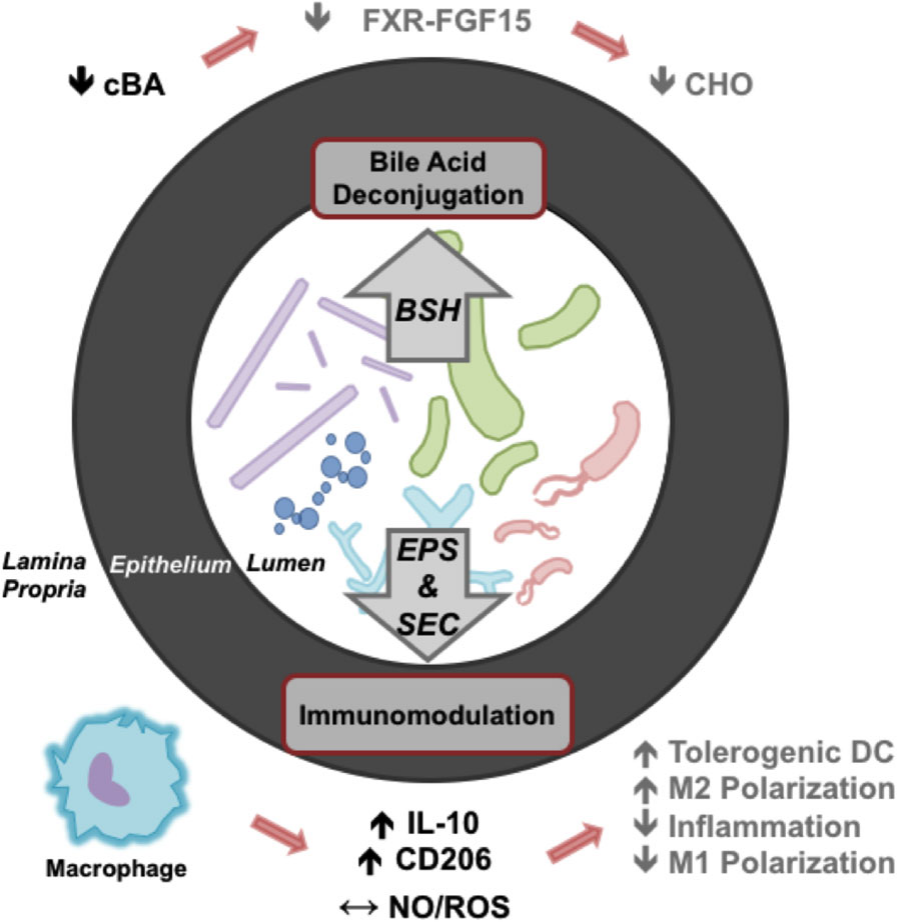
Putative Mechanisms Through Which *Lactobacillus mucosae* DPC 6426 May Exert Cardio-protective Effects. Black text indicates results which are reported in the present study, while grey text represents extrapolations of the potential in vivo effects of such physiological reactions to *Lactobacillus mucosae* DPC 6426 and its metabolites, in the context of cardiovascular disease. cBA, conjugated bile acids; FXR, farnesoid X receptor; FGF15, fibroblast growth factor 15; CHO, cholesterol; BSH, bile salt hydrolase; EPS, exopolysaccharide; SEC, secretome; M1, classically activated macrophage; M2, alternatively activated macrophage; IL-10, interleukin-10; CD206, cluster of differentiation 206; NO, nitric oxide; ROS, reactive oxygen species; DC, dendritic cell

We have, herein, identified two potential mechanisms through which *Lb. mucosae* DPC 6426 may confer cardio-protective effects upon its host (as summarised in Fig. 4). These include one or more bile-modifying enzymes and an EPS capable of immunomodulatory action. Moreover, these data indicate that *Lb. mucosae* DPC 6426 BSH may have a more significant impact on human bile metabolism than that of a rodent, as human bile bears greater resemblance to the *GcBA*-rich profile of porcine bile. This in turn may have further implications for improving human host CVD status, and suggests that BSH activity is perhaps not the main mechanism through which *Lb. mucosae* DPC 6426 attenuates cholesterol accumulation in the apo-E^−/−^ murine model. Furthermore, we have shown that the complex heteropolysaccharide produced by *Lb. mucosae* DPC 6426 can interact with the host immune system in vitro to promote a largely anti-inflammatory tone and factors which are important to endothelial function. Finally, we have previously recorded increased faecal excretion of cholesterol in the apo-E^−/−^ murine model following *Lb. mucosae* DPC 6426, suggesting that the EPS itself may possess some intrinsic sterol-binding capacity. Indeed, each of these strain-specific features may be central to the previously reported cardio-protective attributes associated with *Lb. mucosae* DPC 6426.

## Conclusions

These data suggest that *Lb. mucosae* DPC 6426 has the potential to act on host CVD status through a multitude of pathways. Firstly, the microbe has been shown to express enzymes which shape host bile pool profile in a manner that may promote endothelial function, while also reducing systemic inflammation. This same attribute may in turn alter BA metabolism and signalling in a manner which has potential to improve host lipid and cholesterol metabolism. However, due to the BA specificity of the microbe, it is likely that these effects would only be obvious in the glyco-conjugated BA-rich porcine or human system. In addition, the EPS and secretome produced by *Lb. mucosae* DPC 6426 appears to promote a generally anti-inflammatory tone in vitro, which may contribute to the attenuation of inflammatory-mediated metabolic dysfunctions, if mirrored in vivo.

## Materials & methods

### Bacterial Strains & Culture Conditions

All *Lb. mucosae* strains used in this study - which include DPC 6418, 6420, 6425 and 6426 - are of mammalian intestinal origin and were obtained from the Teagasc Moorepark Food Research Centre culture collection. *Lb. reuteri* APC 2587 is a bile salt hydrolase-active strain of human origin, and was obtained from the APC Microbiome Institute culture collection. RAPD PCR was performed for all strains in this study, using primer sequence 5’-GCTCGTATGTTGTGTGG-3′ as described in Navidghasemizad et al. [53], to ensure genetic uniqueness of closely related strains. All strains were grown from − 80 °C stocks (75% glycerol) on de Mann Rogosa Sharpe (**MRS**; Difco) media solidified with 1.5% agar in gas jars with anaerobic gas packs (Anaerocult A, Merk KGaA, Darmstadt, Germany), at 37 °C for 48 h. Single colonies were subsequently grown overnight and maintained in MRS broth for a maximum of three subcultures prior to assays.

### Exopolysaccharide Isolation & Secretome

EPS was isolated from *Lb. mucosae* DPC 6426 as per López et al. [54]. Fresh culture was autolysed at 100 °C for 15 min in a water bath (Grant, JB series). This suspension was then centrifuged for 30 min at 3000 *x g* (Sorvall RC5B Plus, rotor SLA3000). Supernatant was collected and treated with 10% (*v*/v) 12 M HCl for 5 min at 70 °C, with overhead stirring. The solution was then centrifuged again under the same conditions. Supernatant was again collected and mixed with two volumes of ice-cold ethanol. This mixture was left for 24 h at − 20 °C with mild agitation. Precipitated carbohydrates were collected by centrifugation and resuspended in a minimal amount of ultrapure water. The ethanol precipitation step was repeated twice more. The pelleted carbohydrate was resuspended in minimal ultrapure water and dialyzed with 12–14 kDa MWCO dialysis tubing (Sigma Aldrich, Ireland) against ultrapure water for three days at 4 °C with twice daily water changes. This dialysed solution was then lyophilized (VirTis Advantage Wizard 2.0).

As a secondary purification, the crude EPS was dissolved in 50 mM Tris–HCl, 10 mM MgSO_4_·7H_2_O, pH 7.5 at a final concentration of 5 mg ml^− 1^ and treated with DNAse type-I (Sigma Aldrich, Ireland, final concentration 2.5 μg ml^− 1^) for six hours at 37 °C. The solution was then treated with pronase E (Sigma Aldrich, Ireland, final concentration 50 μg ml^− 1^, dissolved in 50 mM Tris–HCl, 2% EDTA, pH 7.5) for 18 h at 37 °C. Trichloroacetic acid was then added (12% *w*/*v*, final concentration) and stirred at room temperature for 30 min, followed by centrifugation. Resulting supernatant was adjusted to pH 4.0–5.0 with 10 M NaOH. It was then dialyzed against ultrapure water under the previous conditions, followed by lyophilisation.

The secretome which was applied to macrophage in in vitro components of the present study was composed entirely of spent cell-free MRS broth in which *Lb. mucosae* DPC 6426 had been cultured. It is anticipated that this medium contains all the metabolites and enzymes which the strain secretes externally under in vitro culture conditions. Following overnight incubation of the bacteria in broth as previously described, the culture was centrifuged at 22,000 *x g* for 15 min. The resulting supernatant was passed through a 45 μm filter and stored at − 20 °C prior to exposures.

### RAW 264.7 macrophage Secretome & EPS exposures

#### Cell line & culture

The murine macrophage cell line RAW 264.7 (ATCC TIB-71, LCG Systems) was maintained in Dulbecco’s Modified Eagle Medium/Nutrient Mixture F-12/GlutaMAX™ (**DMEM/F-12/GlutaMAX**™; Gibco via Thermo Fisher Scientific) supplemented with 10% heat –inactivated foetal bovine serum (FBS; Sigma-Aldrich) in the presence of 24 ng/ml gentamicin (Gibco via Thermo Fisher Scientific). Experiments and cell culture maintenance were performed at 37 °C in a humidified atmosphere containing 5% CO_2_.

#### Macrophage polarization

RAW 264.7 cells (1 × 10^5^ cells) were seeded in 6-well culture plates and cultured as described above for 48 h. Following 12 h starvation in serum-free medium, control samples for M1 or M2 polarization were stimulated with either 10 ng ml^− 1^ lipopolysaccharide (Sigma-Aldrich) and 50 ng ml^− 1^ mouse IFN-γ (R&D Systems), or 20 ng ml^− 1^ mouse IL-4 (R&D systems) for 24 h, respectively. Unstimulated RAW 264.7 cells were cultivated for 24 h and then exposed to either the secretome of *Lb. mucosae* DPC 6426 or 10 mg ml^− 1^ of purified EPS isolated from the same strain, both suspended in supplemented medium without the antibiotic for 24 h. After washing the samples with supplemented medium, RAW 264.7 cells were harvested and stored at − 80 °C prior to qRT-PCR analysis.

#### Gene transcription in murine macrophage

Total RNA was extracted using RNeasy® (Qiagen) according to the manufactures’ protocol. The RNA concentrations were determined by A_260_ measurements. cDNA synthesis was performed using Tetro cDNA Synthesis Kit (Bioline) with 5 μg RNA per 50 μl reaction, according to manufactures’ instructions. RT-PCR was performed with Lightcycler SYBR Green I MasterKit (Roche Diagnostics Ltd.) and 0.25 mM of the following primers: *Tnf:* 5’AAAT CGGC TGAC GGTG TGGG3’ and 5’CCCA CACC GTCA GCCG ATTT3’; *iNos:* 5’ CAGA GGAC CCAG AGAC AAGC3’ and 5’TGCT GAAA CATT TCCT GTGC3’; *Cd206:* 5’CAGG TGTG GGCT CAGG TAGT3’ and 5’TGTG GTGA GCTG AAAG GTGA3’; *Il-10:* 5’GCTC TTAC TGAC TGGC ATGAG3’ and 5’CGCA GCTC TAGG AGCA TGTG3’; and *Il-6:* 5’CCAG TTGC CTTC TTGG GACT3’ and 5’GGTC TGTT GGGA GTGG TATCC3’. All PCR assays were set up in duplicate and analyzed using the LightCycler 480 system (Roche Diagnostics Ltd.). PCR conditions were set at 10 mins at 95 °C, followed by 50 cycles of 95 °C for 10 s, 60 °C for 5 s and 72 °C for 15 s. *18 s* was used as the reference gene.

### NO measurement and viability/metabolic activity assay

#### Cell line & culture

The macrophage cell line RAW 264.7 (ATCC TIB-71, LCG Systems) was maintained in DMEM as mentioned previously, but in the presence of 100 U/ml penicillin and 100 μg/ml streptomycin (Sigma Aldrich) rather than gentamicin. Cells were passaged twice weekly.

#### Macrophage stimulation

RAW 264.7 cells (1.5 × 10^5^ cells) were seeded in a volume of 200 μl into 96-well culture plates and cultured as described above, except that phenol red was omitted from the medium. The stimulants (different concentrations of EPS isolated from *Lb. mucosae* DPC 6426, with or without the extra addition of 1 μg/ml LPS (Sigma-Aldrich)) were added immediately to the macrophages. After 24 h incubation, 75 μl of supernatant was harvested and stored at − 80 °C for subsequent measurement of secreted cytokines. Another 75 μl of supernatant was transferred to a new plate where NO production was measured using the Griess assay and the remaining culture supernatant and cells were used for a cell viability assay.

#### Nitrite assay

Nitrite, a stable metabolite of NO, produced by activated macrophages was measured by the Griess assay [55]. Briefly, an aliquot of 75 μl culture supernatant from each well was transferred to the wells of a new flat-bottomed 96-well plate and combined with 100 μl of 1% sulfanilamide and 100 μl of 0.1% naphthylenediamine (both were prepared in 2.5% phosphoric acid solution). After 5 min incubation at room temperature, the nitrite concentration was determined by measuring optical density (OD540, reference filter 690 nm) of each well using a SPECTRA MAX microplate reader (Molecular Devices). Sodium nitrite (Sigma Aldrich) was used as a standard to determine nitrite concentrations in the cell-free medium.

#### Viability/metabolic activity assay (XTT)

This assay provides a spectrophotometric method for estimating cell viability based on the mitochondrial activity in living cells, as it measures the mitochondrial dehydrogenase activity of living cells. The mitochondrial dehydrogenases of viable cells reduce the tetrazolium ring of XTT (Sigma Aldrich), yielding an orange formazan derivative, which is water-soluble. Briefly, 20% *v*/v of XTT was added to the wells and the cells were incubated for 3–4 h at 37 °C in a humidified atmosphere containing 5% CO_2_. Subsequently, the amount of formazan derivative was determined by measuring optical density (OD450, reference filter 690 nm) of each well using a SPECTRA MAX microplate reader (Molecular Devices). An increase or decrease in viable cells relative to control cells, results in an accompanying change in the amount of formazan formed.

#### Cytokine production by murine macrophages

To measure IL-10 and TNF-α production, the Ready-Set-Go ELISA (Ebioscience) was used according to the manufacturer’s instructions. Briefly, plates were coated at room temperature for 3 h, after which the plates were blocked for 2 h with blocking buffer. Standards and samples (10 x diluted for TNF-α) were added to the wells and the plates incubated overnight at 4 °C. The following day, the plates were washed by filling and emptying the wells with PBS and then the biotin-conjugated antibody to mouse IL-10 and TNF-α was added for 2 h at room temperature. After washing the plates three times, streptavidin conjugated to horse radish peroxidase was added and incubated for 1 h at room temperature in the dark. After another wash, substrate was added and after allowing for proper colour development, the reaction was terminated using 2 M H_2_SO_4_. Finally, the optical density of the reaction was measured (OD450, reference filter 570 nm) using the SPECTRA MAX microplate reader (Molecular Devices) and compared with the optical density of the known standard samples to determine cytokine concentrations.

#### TLR reporter assay

Human embryonic kidney cells [56] (Invivogen) were transformed with different human Toll like receptors (TLR1/2 [293-mtlr1/2], TLR2 [293-mtlr2] and TLR4 [293-mtlr4]) and a NF-κB luciferase reporter construct (Invivogen). Approximately 6 × 10^4^ cells of each reporter line in DMEM medium supplemented with 100 U/ml penicillin and 100 μg/ml streptomycin (Sigma Aldrich) and 10% fetal bovine serum (Invitrogen) were seeded into wells of a black, clear bottom 96-well plate (Invitrogen) and incubated overnight at 37 °C in a humidified atmosphere containing 5% CO_2_. The cells were subsequently stimulated with different concentrations of EPS isolated from *Lb. mucosae* DPC 6426, PCSK (5 μg/ml, Invitrogen), or different concentrations of LPS (Invivogen) as positive controls or with medium alone (negative control) and incubated for 4 h at 37 °C in a CO_2_ incubator. After this incubation period, half of the medium was replaced with ‘Bright Glow’ (Promega), the plate was then vortexed for 5 min and the luminescence measured using a Spectramax M5 (Molecular Devices). HEK293 cells not expressing any of the TLR receptors but harbouring pNIFTY, a luciferase reporter construct for NF-κB activation (Invivogen, Toulouse, France) were used to control for TLR-independent activation of NF-κB.

### Oxidative burst activity neutrophils

#### Cell line & culture

The human promyelocyte precursor cell line, HL60 (ATCC CCL-240, LCG Systems), was maintained in RPMI medium supplemented with 10% FBS, 100 μg/ml streptomycin, and 100 IU/ml penicillin (Sigma Aldrich) at 37 °C in an atmosphere containing 5% CO_2_. Cell count and viability were performed using a standard trypan blue cell counting technique. To differentiate the cells into neutrophils, cells were grown to a density of 1 × 10^6^ cells/ml with addition of 1.25% dimethyl sulfoxide for three to four days. After washing the cells and re-suspending them in RPMI medium with 10% *v*/v fetal bovine serum (FBS) supplemented with antibiotics, cells displayed typical neutrophilic behaviour and adhered to the plate in less than an hour.

#### Isolation of human neutrophils

Venous blood was drawn from healthy volunteers using vacuette tubes (Greiner Bio-one), coated with lithium heparin. Blood was carefully layered onto a Percoll (GE healthcare) gradient of 1.079 and 1.098 density. After centrifugation for 8 min at 150 *x g* (with minimal brake) and additionally for 10 min at 1200 *x g* (without break), the neutrophils were isolated as a single layer of cells. After lysis of the erythrocytes, the cells were counted and gently resuspended in RPMI medium without phenol red.

#### Nitroblue tetrazolium assay

After 3/4 days of differentiation (HL60), or directly after isolation (freshly isolated neutrophils), cells were seeded at a density of 1.5 × 10^5^ cells/well in RPMI medium without phenol red. After allowing adherence for one hour, plates were centrifuged for 5 min at 750 x *g*, after which the culture supernatant was aspirated and replaced by 153 μl of nitroblue tetrazolium (Sigma Aldrich). Different concentrations of the isolated EPS (1, 5, 10, 50 and 100 μg/ml) were prepared from stock solution by serial dilution in serum-free RPMI medium and 17 μl was added to the nitroblue tetrazolium mixture. For control samples, 17 μl of serum-free RPMI medium was added. The cells were incubated for 90 min at 37 °C in atmosphere containing 5% CO_2_. Following another centrifugation step under the conditions previously mentioned, the nitroblue tetrazolium was aspirated and the cells fixated with 100 μl methanol for 7 min at room temperature. After aspiration of the methanol, the plates were allowed to air dry for 15 min. Finally, 120 μl of KOH and 140 μl of DMSO was added to the plate and thoroughly mixed to completely dissolve the formazan crystals formed by the cells. The amount of formazan derivative was then determined by measuring optical density (OD620, reference filter 414 nm) of each well using a SPECTRA MAX microplate reader (Molecular Devices).

### Bile acid metabolism

#### Bile salt hydrolase plate assay

The ability to deconjugate BAs was determined qualitatively as previously described [57]. Strains were grown under the conditions outlined above at 37 °C for 18 h in MRS_thio_ broth, and 5 μl of this culture was then spotted onto MRS_thio_ plates containing 0.037% *w*/*v* CaCl_2_ and 0.5% w/v either TDCA or GDCA (Sigma Aldrich). Plates were incubated under the conditions described above at 37 °C for 24 h before inspection and BSH activity was deemed present in a strain if a halo was visible surrounding the colony cell mass.

#### Cell-free protein extract preparation

Extracts were prepared from each strain as per the method of Jarocki et al. [58]. Briefly, cultures were grown to stationary phase in 200 ml MRS broth and centrifuged at 10,000 *x g* for 10 min at 4 °C. Pelleted cells were washed twice with PBS (Sigma Aldrich) before being resuspended in 8 ml of PBS with 10 mM 2-mercaptoethanol (Sigma Aldrich). Cell suspensions were then sonicated on wet ice for 3 min followed by centrifugation at 20,000 *x g* for 10 min at 4 °C. The resulting supernatants were stored at − 20 °C prior to assay. The protein content of all extracts was quantified by the Bradford method [59], with bovine serum albumin (Sigma Aldrich) as standard.

#### Bile salt hydrolase activity assay

The extracts were diluted to a protein concentration of approximately 0.5 mg ml^− 1^ in PBS 2-mercaptoethanol and 100 μl of this solution was mixed with 100 μl 20 mM TDCA or GDCA in the same buffer. This suspension was incubated at 37 °C for 30 min, at which point the reaction was terminated by the addition of 200 μl 15% (w/v) trichloroacetic acid (Sigma Aldrich). All proteins were then precipitated out of solution by centrifugation at 14,000 *x g*. The amounts of free amino acids (taurine and glycine) were quantified using a Jeol JLC-500 V AA analyser in conjunction with a Jeol Na^+^ high performance cation exchange column (Jeol Ltd., Tokyo, Japan). One unit of BSH activity is defined as nmol of amino acid cleaved from the relevant BA per mg of protein and expressed per minute (U min^− 1^).

#### Bile deconjugation profiling

The specificity and BA preference of the BSH expressed by *Lb. mucosae* DPC 6426 and by *Lb. reuteri* APC 2587 were assessed in both porcine and murine bile. Fresh overnight cultures were sub-cultured into MRS broth supplemented with 0.5% of either porcine or murine gall bladder bile. Samples were prepared and analysed for BA profile by the adjusted UPLC-MS method of Swann et al. [60], described previously in Joyce et al. [10].

### Statistical analysis

All RT-PCR cell culture experiments were conducted three times with duplicate samples. BSH-activity and individual bile fluctuations in bile deconjugation profiling results were analysed by one-way ANOVA with a Bonferroni correction for multiple comparisons. For all statistical tests, differences between means were considered significant if the *P* value was < 0.05.

## Additional file

Additional file 1: Document Contains Information on Bacterial Strain Origins, as well as Data on Neutrophil Reactive Oxygen Species Assays and Bile Deconjugation Profiles. **Figure S1.** Relative Oxidative Burst Activity Of Differentiated HL-60 Human Neutrophils and Freshly Isolated Human Neutrophils. Neutrophils were stimulated for 90 min with or without additional PMA stimulation after which reactive oxygen species production was measured. Experiments were often repeated and are depicted as reactive oxygen production relative to medium control (A; HL-60 cells, C; freshly isolated neutrophils) or relative to PMA (1 ng.ml^− 1^) stimulated samples (B; HL-60 cells, D; freshly isolated neutrophils from two donors). Data are shown as average ± SD. Differences were considered statistically significant when *p* < 0.05 (*), *p* < 0.01 (**) or *p* < 0.001 (***). **Table S1.** Origins of Strains. **Table S2.** Full Bile Deconjugation Experiment Profile. Full quantitative list of bile acids in MRS supplemented with murine or porcine bile untreated, or cultured with APC 2587 or DPC 6426. Data represent the means of duplicate experiments for untreated bile, and triplicates for cultured bile. Means with the * postfix are significantly different (*p* < 0.05) from the untreated bile concentration, as assessed by one-way ANOVA. (DOCX 115 kb)

## Abbreviations

BA: bile acid
BSH: bile salt hydrolases
CA: cholic acid
CDCA: chenodeoxycholic acid
CVD: Cardiovascular disease
DCA: deoxycholic acid
DCA: deoxycholic acid
EPS: exopolysaccharides
FGF: fibroblast growth factor
FXR: farnesoid X receptor
GcBA: glyco-conjugated BA
GDCA: glycodeoxycholic acid
GHDCA: glycohyodeoxycholic acid
GLP-1: glucagon-like peptide-1
IFN-γ: interferon-γ
IL: interleukin
iNOS: inducible nitric oxide synthase
LCA: lithocholic acid
LPS: lipopolysaccharide
MRS: de Mann Rogosa Sharpe
NF-κB: nuclear factor-κB
pBA: primary bile acids
PCA: Principle component analysis
ROS: reactive oxygen species
sBA: secondary bile acids
TcBA: tauro-conjugated bile acids
TDCA: taurodeoxycholic acid
TLCA: taurolithocholic acid
TLR: Toll-like receptor
TNF-α: tumor necrosis factor-α
