# Supplementary material for: Lactobacillus mucosae DPC 6426 as a bile-modifying and immunomodulatory microbe
Source: BMC Microbiol. 2019 Feb 8;19:33. doi: 10.1186/s12866-019-1403-0 (PMC6368806; doi:10.1186/s12866-019-1403-0)
Supplement: Supplementary file 1 — Document Contains Information on Bacterial Strain Origins, as well as Data on Neutrophil Reactive Oxygen Species Assays and Bile Deconjugation Profiles. Figure S1. Relative Oxidative Burst Activity Of Differentiated HL-60 Human Neutrophils and Freshly Isolated Human Neutrophils. Neutrophils were stimulated for 90 min with or without additional PMA stimulation after which reactive oxygen species production was measured. Experiments were often repeated and are depicted as reactive oxygen production relative to medium control (A; HL-60 cells, C; freshly isolated neutrophils) or relative to PMA (1 ng.ml− 1) stimulated samples (B; HL-60 cells, D; freshly isolated neutrophils from two donors). Data are shown as average ± SD. Differences were considered statistically significant when p < 0.05 (*), p < 0.01 (**) or p < 0.001 (***). Table S1. Origins of Strains. Table S2. Full Bile Deconjugation Experiment Profile. Full quantitative list of bile acids in MRS supplemented with murine or porcine bile untreated, or cultured with APC 2587 or DPC 6426. Data represent the means of duplicate experiments for untreated bile, and triplicates for cultured bile. Means with the * postfix are significantly different (p < 0.05) from the untreated bile concentration, as assessed by one-way ANOVA. (DOCX 115 kb) [file 12866_2019_1403_MOESM1_ESM.docx]

**Supplementary Data**


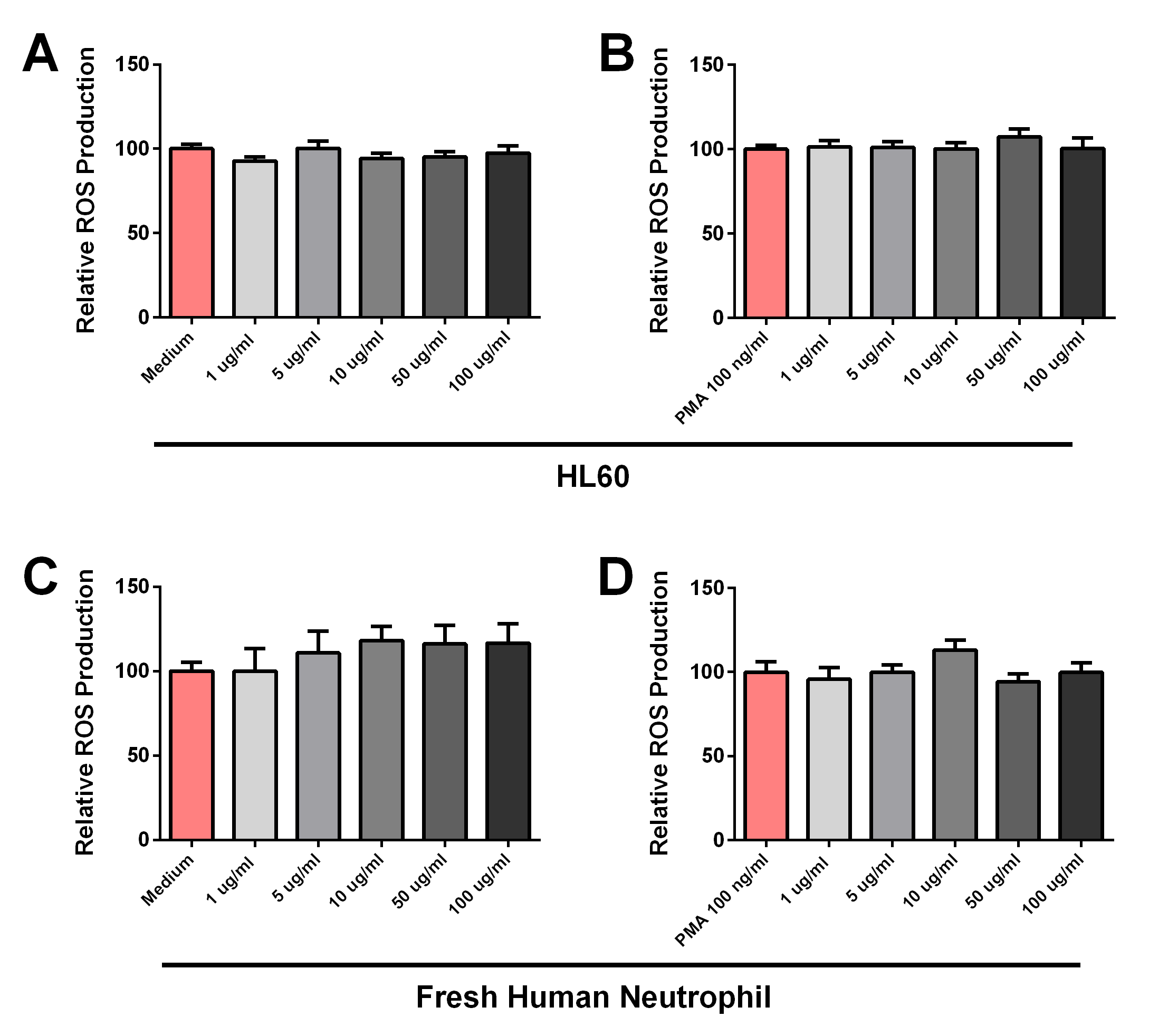


**Additional file 1: Figure S1 – Relative Oxidative Burst Activity Of Differentiated HL-60 Human Neutrophils and Freshly Isolated Human Neutrophils.** Neutrophils were stimulated for 90 minutes with or without additional PMA stimulation after which reactive oxygen species production was measured. Experiments were often repeated and are depicted as reactive oxygen production relative to medium control (A; HL-60 cells, C; freshly isolated neutrophils) or relative to PMA (1 ng.ml^-1^) stimulated samples (B; HL-60 cells, D; freshly isolated neutrophils from two donors). Data is shown as average ± SD. Differences were considered statistically significant when p < 0.05 (*), p < 0.01 (**) or p < 0.001 (***).

**Additional file 1: Table S1 – Origins of Strains.**

| Species | Strain | Origin |
| --- | --- | --- |
| *Lactobacillus reuteri* | APC 2587 | Human |
| *Lactobacillus mucosae* | DPC 6418 | Bovine |
|  | DPC 6420 | Bovine |
|  | DPC 6425 | Bovine |
|  | DPC 6426 | Bovine |

Additional file 1: Table S2 – Full Bile Deconjugation Experiment Profile. Full quantitative list of bile acids in MRS supplemented with murine or porcine bile untreated, or cultured with APC 2587 or DPC 6426. Data represent the mean ng/ml. Italics indicates significant different (p < 0.05) from the untreated bile concentration.
